# Effects of benzoic acid *(VevoVitall®)* on the performance and jejunal digestive physiology in young pigs

**DOI:** 10.1186/s40104-016-0091-y

**Published:** 2016-05-28

**Authors:** Hui Diao, Zengbing Gao, Bing Yu, Ping Zheng, Jun He, Jie Yu, Zhiqing Huang, Daiwen Chen, Xiangbing Mao

**Affiliations:** Animal Nutrition Institute, Sichuan Agricultural University, Xinkang Road 46#, Ya’an, Sichuan Province 625014 People’s Republic of China

**Keywords:** Benzoic acid, Glucagon-like peptide 2, Nutrient digestibility, Performance, Young pigs

## Abstract

**Background:**

As a organic acid, benzoic acid has become one of the most important alternatives for antibiotics, and its beneficial effect on performance in animals has been proven for a decade. However, knowledge of the effects of benzoic acid on jejunal digestive physiology, especially the antioxidant capacity and mucosal glucagon-like peptide 2 (GLP-2) concentrations is lacking.

**Methods:**

A total of 20 barrows [Duroc × (Yorkshire × Landrace)] with an average body weight (BW) of 18.75 ± 0.2 kg were used in a 14-d trial to determine the potential mechanisms of benzoic acid supplementation on the performance, nutrient digestibility and jejunal digestive physiology in young pigs. All pigs were randomly allotted to 1 of 2 diets supplemented with 0 or 5000 mg/kg benzoic acid.

**Results:**

Relative to the control, benzoic acid supplementation increased the average daily feed intake (ADFI), and average daily gain (ADG) in young pigs (*P* < 0.05), improved the apparent total tract digestibility of dry matter (DM), crude protein (CP), ether extract (EE), gross energy (GE) and crude ash (*P* < 0.05), and enhanced the activities of trypsin, lipase and amylase in the jejunum (*P* < 0.05). Similarly, relative to the control, supplementing benzoic acid in the diet resulted in a trend to reduce the pH values of the digesta (*P* = 0.06), decreased crypt depth and increased the villus height to crypt depth ratio (*P* < 0.05) in the jejunum of pigs. Finally, benzoic acid supplementation increased the mRNA expression and concentration of glucagon-like peptide 2 and the activities of glutathione peroxidase and superoxide dismutase in the jejunal mucosa of young pigs (*P* < 0.05).

**Conclusions:**

In conclusion, supplementation with 5000 mg/kg benzoic acid improved the performance of young pigs through promoting nutrient digestion, improving jejunal antioxidant capacity, and maintaining the jejunal morphology in young pigs.

## Background

To overcome the negative effect on pig production brought by the ban of antibiotics in Europe, using different alternatives, such as organic acids and essential oils, has been recommended as one of the effective methods to help improve the performance and decrease the incidence of diarrhea in pigs [1]. As the simplest of the aromatic carboxylic acids, benzoic acid has broad-spectrum antimicrobial properties and forms colourless to white crystals, and was authorized to be used for the preservation of various foodstuffs and feed of growing pigs at the dose of 0.5 to 1.0 % by the European Union in 2003 [2].

Previous studies have shown that benzoic acid can improve the performance and nutrient digestibility, inhibit pathogenic microorganisms, and maintain the balance of microflora [3–7]. However, there is little available information on the systematic mechanisms through which benzoic acid promotes pig performance, especially the effect of benzoic acid on the antioxidant capacity and mucosal glucagon-like peptide 2 (GLP-2) concentrations in the jejunum of pigs. Thus, the objective of this study was to evaluate the effects of benzoic acid on the performance, nutrient digestibility, pH values, activities of digestive enzymes, mucosal morphology, GLP-2 concentration and antioxidant capacity in the jejunal mucosa of young pigs, which could help us further understand the mechanism through which benzoic acid improves pig performance, and provide the scientific basis for using benzoic acid in practice.

## Methods

The experimental protocol used in this study was approved by the Animal Care and Use Committee of Sichuan Agricultural University (Ya’an, China). Benzoic acid (*VevoVitall*) was provided by DSM (China) Limited (Shanghai, China).

### Experimental design, animals and diet

A total of 20 healthy DLY [Duroc × (Yorkshire × Landrace)] young pigs with an initial average BW of 18.75 ± 0.20 kg were randomly allotted to 1 of 2 diets including a control and a diet supplemented with 5000 mg/kg benzoic acid for 14 days During d 11-14, the apparent total tract digestibility (ATTD) of nutrients was measured using the acid insoluble ash (AIA) as an endogenous indicator. All pigs were placed individually in metabolism cages (1.5 m × 0.7 m × 1.0 m) with a self-feeder and a nipple watering device, which were located in a temperature (25 ± 1 °C) and relative humidity (60 ± 5 %)-controlled room. The pigs had free access to feed and drinking water. The lighting was natural. Individual BW was recorded after all pigs were food-deprived for 12 h on d 1 and 15, and feed consumption was recorded as the amount of feed offered daily minus the remaining quantity in the next morning during the experiment, which were used to determine the average weight gain (ADG), average daily feed intake (ADFI) and ratio of feed to gain (F/G).

The diets were formulated to meet or exceed the National Research Council recommended nutrient requirements for pigs weighing 10-20 kg (NRC, 2012) [8]. Ingredients and composition of the diets is presented in Table 1. The experimental diet was formulated using benzoic acid to substitute for maize in the basal diet. No antibiotics were used in any diet.

**Table 1:** Ingredients and nutrient compositions of basal diet, air dry basis,%

Ingredients,%		Calculated Composition,%		Analyzed Composition,%	
Maize	57.88	DE,MJ/kg	14.37	Control diet	
Dehulled soybean meal	22.00	Available phosphorus	0.41	Crude protein	18.32
Fish meal	5.00	Arg	1.20	Calcium	0.86
Soybean oil	1.50	Cys	0.32	Total phosphorus	0.62
Whey powder	6.00	His	0.49	Ether extract	0.73
Sugar	2.00	Ile	0.80	Gross energy,kcal/g	4.17
Glucose	3.00	Leu	1.65	Benzoic acid,mg/kg	0
*L-*Lys-HCl,78 %	0.24	Lys	1.31	Benzoic acid diet	
*DL*-Met,99 %	0.10	Met	0.45	Crude protein	18.36
Trp,98 %	0.02	Met + Cys	0.77	Calcium	0.83
Thr,98.5 %	0.07	Phe	0.91	Total phosphorus	0.59
Choline Chloride	0.10	Thr	0.84	Ether extract	0.72
Calcium carbonat	0.90	Trp	0.25	Gross energy,kcal/g	4.19
Dicalcium phosphate	0.40	Na	0.24	Benzoic acid,mg/kg	5036
Nacl	0.15				
Vitamin Complex^a^	0.04				
Mineral Complex^b^	0.30				
Phytase	0.10				

^a^The premix provides following per kg diet: Vitamin A 6000 IU; Vitamin D_3_ 400 IUg; Vitamin E 10 IU; Vitamin K_3_ 3 mg; Vitamin B_1_ 3 mg; Vitamin B_2_ 6 mg; Vitamin B_6_ 3 mg; Vitamin B_12_ 24 μg; folic acid 1.2 mg; nicotinic acid 14 mg; biotin 150 mg; D-pantothenic acid 15 mg

^b^The premix provides following per kg diet: Fe (as ferrous sulfate) 100 mg, Cu (as copper sulfate) 6 mg, Mn 40 mg, Zn (as zinc sulfate) 80 mg, I 0.3 mg, Se 0.3 mg

### Sampling and measurements

Experimental diets were sampled and stored at -20 °C until analyzed for dry matter (DM), crude protein (CP), ether extract (EE), gross energy (GE) and crude ash. After each collection of feces, 10 % hydrochloric acid was added to fix excreta nitrogen. Feces collected on d 11-14 of each replicate were dried in a forced air oven (60 °C) for 72 h, and ground through a 1-mm screen before chemical analysis.

On d 15, following weighing, all the pigs were killed with an intravenous injection of chlorpromazine hydrochloride (Shanghai Harvest Pharmaceutical Co. Ltd. China, 150 mg/kg body weight) and jugular exsanguinations. Then, the mid-jejunum (2 cm) was immediately isolated, washed and preserved in 10 % formalin solution for histological analysis. This was followed by measuring the pH of the jejunal digesta with a pH meter (PHS-3C pH, Shanghai, China). The jejunal mucosa were collected by scraping the intestinal wall with a glass microscope slide, immediately frozen in liquid nitrogen, and stored at -80 °C until the analysis of the antioxidant capacity, GLP-2 concentration and quantitative real-time PCR. Additionally, the jejunal digesta was collected, and stored at -80 °C for measuring the activities of the digestive enzymes.

### Apparent total tract digestibility

The ATTD was measured using AIA as a digestibility indicator. AIA in diets and fecal samples were determined by a method described by the Chinese National Standard (GB/T 23742) [9]. All diets and fecal samples were analyzed for DM (method 930.15, AOAC, 1995), ash (method 942.05, AOAC, 1995), EE (method 945.16, AOAC, 1995), Ca (method 927.02, AOAC, 1995), P (method 995.11, AOAC, 1995), CP (method 990.03, AOAC, 1995) [10] and GE. The GE concentration was determined using bomb calorimetry (Parr Instrument 1563, Moline IL). The content of benzoic acid in the diets was measured by DSM (China) Limited (Shanghai, China) using high-performance liquid chromatography. The digestibility of chemical constituents was calculated as (100 – A1/A2 x F2/F1 x 100), where A1 represents the AIA content of the diet; A2 represents the AIA content of the feces; F1 represents the nutrient content of the diet; F2 represents the nutrient content of the feces [11].

### Histological measurements

The measurements of the villous height and crypt depth were conducted as described by Pluske *et al* [12]. Briefly, the ring-shaped histological sections of the jejunum were excised, dehydrated, and embedded in paraffin wax before 4 transverse sections (5 μm) were cut, and then installed on glass slides and stained with haematoxylin and eosin. The height of 10 well orientated villi and their adjoined crypts were measured at 40 × magnification with an Olympus CK 40 Microscope (Olympus Optical Company, Guangzhou, China).

### Digestive enzymes activities, antioxidant capacity and GLP-2 concentration

The activities of digestive enzymes (trypsin, lipase and amylase) in frozen jejunal digesta, the activities of superoxide dismutase (SOD) and glutathione peroxidase (GSH-px) and the methane dicarboxylic aldehyde (MDA) concentration in the jejunal mucosa were determined using commercial kits obtained from Nanjing Jiancheng Bioengineering Institute (Nanjing, China) combined with a UV-VIS Spectrophotometer (UV1100, MAPADA, Shanghai, China) according to the manufacturer’s instructions.

The GLP-2 concentration of the jejunal mucosa was determined using a commercially available pig Enzyme-linked Immunosorbent Assay Kit from R&D system (Minneapolis, MN) according to the manufacturer’s instructions. The GLP-2 concentration was quantified using a BioTek Synergy HT microplate reader (BioTek Instruments, Winooski, VT), and absorbance was measured at 450 nm.

### Total RNA extraction and reverse transcription reaction

Total RNA was isolated from the frozen jejuna mucosa (one sample per replicate) using the TRIzol reagent (Biotechnology Company, Dalian, China) according to the manufacturer’s protocol. The concentration and purity of total RNA were analysed on a spectrophotometer (Beckman Colter DU 800, Beckman Coulter Inc, Brea, USA), and the optical density 260:280 (OD260:OD280) ranged from 1.8 to 2.0 for all the samples. The synthesis of the first strand of cDNA of each sample was obtained by reverse transcription by RT Reagents (Biotechnology Company, Dalian, China) according to the manufacturer’s instructions.

### Real-time quantitative PCR

Real-time quantitative PCR was carried out using the CFX96 Real-Time PCR Detection System (Bio-Rad Laboratories, Richmond, CA), as described by Mao et al. (2014) [13]. Briefly, the gene-specific primers used in the present study were synthesised commercially by Invitrogen (Shanghai, China), which are listed in Table 2. The PCR system was composed of 5 μL 2 × SYBR Premix Ex Taq (Biotechnology Company, Dalian, China), 0.5 μL of forward and 0.5 μL of reverse primers (100 nmol/L), 3 μL diethylpyrocarbonate-treated water and 1 μL cDNA. Cycling conditions were as follows: 95 °C for 10 s, followed by forty cycles of 95 °C for 5 s, annealing at 62 °C for 10 s and 72 °C for 15 s. The melting curve conditions were 95 °C for 30 s, 55 °C for 1 min and 95 °C for 1 min, which was carried out after each real-time quantitative PCR to check and verify the specificity and purity of all PCR products. Each sample was run simultaneously in triplicate on the same PCR plate, and the average of each triplicate value expressed as the number of copies was used for the statistical analysis. A standard curve was established using serial dilutions of one of the complementary DNA samples, which could be used for obtaining reliable amplification efficiency values (ranged from 90 to 110 %). β-actin was chosen as the reference gene to normalize the mRNA concentration of the target gene, which was calculated with the previous method [14].

**Table 2:** Primes for real time PCR

Target gene	Forward primer 5’-3’	Reverse primer 5’-3’	Accession number
*GLP-2*	TGTAATGCTGGTACAAGGCAG	CTTGTCTTCAGTCATCTGATC	NM_214324.1
*β-actin*	TCTGGCACCACACCTTCT	TGATCTGGGTCATCTTCTCAC	DQ178122

### Statistical analysis

Data were analyzed by *T*-test using the statistical program of SAS version 9.0 (SAS Inst. Inc., NC) where each pig was the statistical unit. All differences were considered significant at *P* < 0.05 and *P* values between 0.05 and 0.10 were considered a trend.

## Results

### Performance

When compared with the control group, 5000 mg/kg benzoic acid supplementation significantly increased ADFI, ADG and final BW of pigs (Table 3, *P* < 0.05). However, no differences were observed among dietary treatments in G/F.

**Table 3:** Effect of benzoic acid on growth performance in young pigs

Items	Control	Benzoic acid	SEM	*P*-value
Initial weight,kg	18.74	18.75	0.07	0.974
14 d weight,kg	27.02^b^	28.79^a^	0.12	0.004
Average daily gain,g	595^b^	718^a^	8.01	0.012
Average daily feed intake,g	1035^b^	1190^a^	12.22	0.005
Feed: gain ratio	1.76	1.66	0.02	0.204

Mean values with their standard errors, *n* = 10

^a-b^ Within a row, means with different superscripts differ (*P* < 0.05)

### Apparent total tract digestibility

As shown in Table 4, the ATTD of CP, DM, EE, GE and ash in pigs fed the benzoic acid diet was greater than that in pigs fed the control diet (*P* < 0.05).

**Table 4:** Effect of benzoic acid on nutrient digestibility in young pigs

Items	Control	Benzoic acid	SEM	*P*-value
Crude protein	0.901^b^	0.925^a^	0.003	0.006
Dry matter	0.916^b^	0.942^a^	0.002	<0.001
Ether extract	0.851^b^	0.900^a^	0.008	0.011
Gross energy	0.918^b^	0.945^a^	0.002	<0.001
Ash	0.732^b^	0.808^a^	0.005	<0.001

Mean values with their standard errors, *n* = 10

^a-b^ Within a row, means with different superscripts differ (*P* < 0.05)

### Digestive enzymes activities and pH values in jejunal digesta

When compared with the control group, 5000 mg/kg benzoic acid supplementation tended to decrease the pH value of the jejunal digesta of pigs (*P* < 0.10, Table 5), and significantly increased the activities of trypsin, lipase and amylase in the jejunal digesta of pigs (*P* < 0.05).

**Table 5:** Effect of benzoic acid on pH values and digestive enzyme activities in jejunum of young pigs

Items	Control	Benzoic acid	SEM	*P*-value
pH	6.2	5.7	0.11	0.064
Trypsin,U•10^3^/mg protein	45.3^b^	48.2^a^	0.64	0.039
Lipase,U•10^3^/g protein	2.7^b^	2.9^a^	0.32	0.028
Amylase,U/mg protein	309.1^b^	401.9^a^	15.81	0.030

Mean values with their standard errors, *n* = 10

^a-b^ Within a row, means with different superscripts differ (*P* < 0.05)

### Histological measurements

When compared with the control group, 5000 mg/kg benzoic acid supplementation significantly increased the villous height to crypt depth ratio, and decreased crypt depth in the jejunum of pigs (*P* < 0.05, Table 6). No differences were observed for the villous height in the jejunum between the two goups.

**Table 6:** Effect of benzoic acid on jejunal morphology in young pigs

Items	Control	Benzoic acid	SEM	*P*-value
Villus height,μm	178	198	4.15	0.394
Crypt depth,μm	162^b^	129^a^	3.16	0.046
Villus height:crypt depth	0.94^b^	1.40^a^	0.03	0.019

Mean values with their standard errors, *n* = 10

^a-b^ Within a row, means with different superscripts differ (*P* < 0.05)

### GLP-2 concentration and mRNA expression in jejunal mucosa

As shown in Table 7, 5000 mg/kg benzoic acid supplementation significantly increased the concentration and mRNA expression of GLP-2 in the jejunal mucosa of pigs (*P* < 0.05).

**Table 7:** Effect of benzoic acid on Glucagon-like peptide 2 concentration and gene relative expression in the jejunal mucosa of young pigs

Items	Control	Benzoic acid	SEM	*P*-value
Glucagon-like peptide 2 concentration,pmol/L	4.12^b^	6.66^a^	0.17	<0.001
Glucagon-like peptide 2 gene relative expression	1.00^b^	1.63^a^	0.04	0.011

Mean values with their standard errors, *n* = 10

^a-b^ Within a row, means with different superscripts differ (*P* < 0.05)

### Enzymes activities of SOD, GSH-PX and MDA concentration

When compared with the control group, 5000 mg/kg benzoic acid supplementation significantly increased the activities of SOD and GSH-PX in the jejunal mucosa of pigs (*P* < 0.05, Table 8). There were no difference or the MDA concentration in the jejunal mucosa between the two groups.

**Table 8:** Effect of benzoic acid on enzyme activities of SOD, GSH-px and concentration of MDA in jejunal mucosa of young pigs

Items	Control	Benzoic acid	SEM	*P*-value
Superoxide Dismutase,U/mL	29.07^b^	70.43^a^	5.73	0.011
Glutathione peroxidase,U/mg	83.08^b^	345.97^a^	22.88	0.049
Malondialdehyde,μmol/L	1.71	1.52	0.09	0.322

Mean values with their standard errors, *n* = 10

^a-b^ Within a row, means with different superscripts differ (*P* < 0.05)

## Discussion

Many studies have recently focused on alternatives to antibiotics, and benzoic acid has been proposed as a feed additive for benefiting animal health. An in vitro study has shown that benzoic acid had the strong antibacterial action, which provided the basis to its application in animals [15]. Recent studies have shown that, 5000 mg/kg benzoic acid supplementation improved the performance in weaned or nursery pigs [3, 4, 16–18], which is similar with this study (Table 3). The effect of benzoic acid on the performance of pigs may be at least partly associated with an improvement of dietary pH and dietary buffering capacity [19], which can enhance the dietary digestibility, increase the microbial diversity of the intestine [3], control the growth of pathogenic bacteria, and establish a proper balance between beneficial and pathogenic microbes in the gastrointestinal tract [16]. However, Kluge et al. reported that 5000 mg/kg benzoic acid supplementation in the diet had no effect on the performance in weaned pigs, while 10,000 mg/kg benzoic acid supplementation significantly improved the performance [5]. These different results of benzoic acid affecting the growth of pigs may be derived from the benzoic acid purity in the different sources and manufacturers, as well as the differences in the age and breed of pigs, the nutrient composition of the diet or the environmental conditions.

Previous studies have shown that diet supplementation with a single acid or compound acidifiers could decrease pH values, and increase the activities of digestive enzymes in the gastrointestinal tract [20–23]. In addition, our previous studies have also shown that 5000 mg/kg benzoic acid supplementation could decrease pH value, and increased the activities of trypsin, lipase, amylase, maltase, sucrase and lactase in the jejunal digesta of weaned pig [7, 24], which was consistent with this study. Therefore, it is possible that benzoic acid supplementation increased the digestive ability of pigs. This study also showed that diet supplementation with 5000 mg/kg benzoic acid could enhance the ATTD of nutrients, which is consistent with the previous studies [16, 25]. However, Galassi et al. [26] reported that 5000 mg/kg benzoic acid supplementation had no effect on the nutrient ATTD in growing pigs (the last phase of growth). The different results could be due to the different physiological stages of experimental pigs. Thus, it could be proposed that benzoic acid supplementation improving the digestive ability of pigs would be weakened as the pigs age.

The jejunum is the key tissue for nutrient digestion, absorption and transportation, and the function of digestion and transportation mainly depends on the villous in the jejunum [27]. Therefore, whether the mucosa structure state is adequate or not is related to nutrient digestion, absorption and growth of the animal. Halas et al. [17] demonstrated diet supplementation with 5000 mg/kg benzoic acid improved intestinal morphology (higher villous height and the villous height to crypt depth ratio), and thus improved nutrient absorption and performance of piglets, which was consistent with this study. The reduction of crypt depth represents greater mature epithelial cells, which results in stronger secretion function, and the higher villous height and villous height to crypt depth ratio indicates more favorable mucosa structure and larger absorptive area of total luminal villous, which could result in adequate digestive enzyme development and better digestibility [12]. Our study showed benzoic acid improved mucosa structure state, along with bringing about superior nutrient digestion and growth in pigs. Previous studies reported that the improved morphology in digestive tract was related to a more acidic environment [28] and higher concentration of volatile fatty acids [16] and larger sucrase and lactase activities in the striated border [29], which could give rise to promoting cell growth and division through stabilizing DNA and repair damage [30], and these factors may contribute to the better morphology in the jejunum brought about by benzoic acid.

GLP-2, a intestinal nutrition factor, could promote the protein synthesis, weight and villous height of the small intestine, increase nutrient digestion and absorption, and improve the barrier function and development of the intestine [31–33]. Its beneficial effect on the intestine could be due to the fact that GLP-2 may decrease the apoptosis of epithelial cells, increase cell proliferation in intestinal mucosa, and promote the growth and regenerative repair after injury of intestinal mucosa [31, 34]. However, in our study, 5000 mg/kg benzoic acid supplementation increased the GLP-2 concentration and relative gene expression in the jejunal mucosa, which verified that benzoic acid could improve the morphology of the jejunum via upregulating the GLP-2 concentration in the intestine of pigs. Moreover, in our previous in vitro study on intestinal porcine epithelial cells (IPEC-1) we revealed a consistent result, which showed 10 μg/mL benzoic acid promoted growth of the jejunum epithelial cells IPEC-1 and up-regulated the relative expression of GLP-2 (unpublished).

In the normal physiological condition, the digestive tract can produce reactive oxygen species (ROS) [35]. In response to the injury of free radicals, there are two antioxidant systems which existe in the body, namely, the enzymatic and non-enzymatic antioxidant system, and enzymatic antioxidant system mainly consists of SOD and GSH-px [36]. SOD can disproportionate O^2−^ into H_2_O_2_, and GSH-px can transform lipid hydroperoxide into alcohol and block lipid peroxidation reaction, which could prevent ROS from reacting with protein, lipid, nucleic acid, carbohydrates or other molecules, and resist the denaturation of these molecules, and thus decrease inflammation, apoptosis and maintain mucosal integrity [36]. In this study, 5000 mg/kg benzoic acid supplementation increased the activities of SOD and GSH-PX in the jejunal mucosa of pigs, which could increase the antioxidant capacity, and then improve the morphology of the jejunum. However, there have been no other reports relating to the effects of benzoic acid on antioxidant capacity in animals, and the mechanism remains unclear, thus, further research is clearly warranted.

## Conclusion

In conclusion, diet supplementation with 5000 mg/kg benzoic acid improved the performance of young pigs at least partly through improving the nutrient digestion and the jejunal morphology. Moreover, benzoic acid supplementation improved the intestinal function possibly via increasing the GLP-2 production and the antioxidant capacity in the intestine of young pigs.
